# A Richer Map for Searching Scientific Literature

**DOI:** 10.1371/journal.pbio.0020343

**Published:** 2004-09-21

Anyone who has used a search engine quickly becomes familiar with both their power and limitations. A key-word search for “bush kerry butterfly county” turns up almost twenty-eight thousand documents, but a scant few of them are of any interest to the botanist studying the butterfly bush in County Kerry. The problem is more circumscribed but no less significant within specialized databases, such as the fourteen-plus million medical journal articles catalogued by PubMed.

It is not so much that the literature is too vast, but that the search strategies are too weak. A simple list of key words used to tag and retrieve a document cannot begin to capture the richness of the information within, especially when that wealth is expressed in syntactically complex sentences like “It has not escaped our notice that the specific pairing we have postulated immediately suggests a possible copying mechanism for the genetic material.”

But there is another way. Rather than simply extracting a limited list of key words from an article's abstract, the entire text of the document can be categorized into classes: some words represent entities (e.g., gene, cellular component, molecular function) and others relationships (e.g., physical association, purpose, comparison, regulation). The entire set of entities and relationships can be linked to create a map of the information within the document, which, like a physical map, captures some of the complexity of the territory it describes.

Humans excel at this type of concept mapping, but their labors are slow and expensive. In this issue, Paul Sternberg and colleagues at the California Institute of Technology (Caltech) describe a computer-based system that performs the same task, and show that it is almost as good as humans at mapping out the scientific literature concerning the laboratory nematode, Caenorhabditis elegans.

Sternberg's system, called Textpresso, includes 33 categories of terms, both of entities and relationships, and a full list of all possible examples for each entity (for genes, for example, this would be specific gene names) and relationship (for physical association, this would include bind, adhere, link, etc.). This collection, called an ontology, is then applied to sentences within the text of a document to map out the relationships within—for instance, the mention of two genes within a sentence along with any form of the word stem “regulat-” indicates that one gene probably regulates the other, and the sentence is marked accordingly. With scores of tags applied, the full markup of a sentence is typically much longer than the sentence itself. Currently, Textpresso has marked up almost 4,000 full-text articles on C. elegans, representing 60% of the entire literature.

**Figure:**
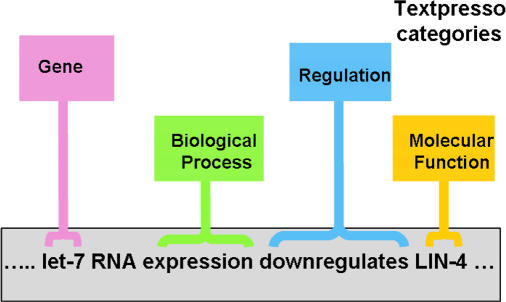
Identifying terms in scientific prose

The final result is a document that can be queried in subtle ways impossible with mere key words. For instance, to find entities (whether they be transcription factors, small molecules, or anything else described to date) that interact with the aging-related gene *daf-16*, one enters the terms “daf-16” and “association.” Textpresso returns 125 publications, with citations and links to the articles. (Textpresso is available at www.textpresso.org.) The results can be further refined by adding other entities or relationships, as well as by specifying author, journal, or year of publication.

Textpresso's ability to find relevant documents, and ignore irrelevant ones, is still not as great as an expert human curator of the same literature. But the system can be constantly tweaked to get better and better. This process requires human intervention, and the Caltech team does not think this is likely to be automated anytime soon. On the other hand, the structure of Textpresso, and to some extent the ontological lists from C. elegans, can be used for literature analysis of other model organisms. Finally, the fully annotated literature within a field is not only a repository of scientific facts, but also a data mine of human communication, which can be queried for patterns having little to do with model organisms and much to do with how scientists communicate with each other.

